# Predictive value of the characteristics of intraretinal cystoid spaces on early response to antivascular endothelial growth factor treatment in patients with cystoid diabetic macular edema

**DOI:** 10.5935/0004-2749.2021-0462

**Published:** 2023

**Authors:** Eyyup Karahan, Omer Can Kayikcioglu, Gozde Sahin Vural, Cenap Guler

**Affiliations:** 1 Department of Ophthalmology, Balıkesir University Faculty of Medicine, Balıkesir, Turkey

**Keywords:** Diabetes Mellitus, Diabetic retinopathy, Macular edema, Tomography, optical coherence, Intraretinal cystoid spaces, Visual acuity, Diabetes Mellitus, Retinopatia diabética, Edema macular, Tomografia de coerência óptica, Espaços cistoides intrarretinianos, Acuidade visual

## Abstract

**Purpose:**

To evaluate whether baseline spectral-domain optical coherence tomography characteristics of intraretinal cystoid spaces predict visual outcomes in patients receiving intravitreal antivascular endothelial growth factor injection therapy (bevacizumab 1.25mg/0.05ml) for diabetic cystoid macular edema.

**Methods:**

The relationship between the properties of the cystoid spaces before injection and anatomic and functional results after injection were investigated in patients who received three consecutive intravitreal bevacizumab injections for cystoid macular edema. The best-corrected visual acuity for functional success and central subfield thickness for anatomical success were evaluated. The relationship of the location of the cystoid spaces with the integrity of photoreceptors and inner retinal layers was also evaluated.

**Results:**

In 36 eyes of 36 patients, the mean best-corrected visual acuity significantly improved (p=0.002), and mean central subfield thickness decreased after injections (p=0.003). The improvement in best-corrected visual acuity was limited in eyes with outer nuclear layer cysts (p=0.045). Intracystic reflectivity was higher in eyes that poor best-corrected visual acuity than in eyes with successful visual outcomes (p=0.028). The disrupted ellipsoid zone was present in 13 (59.0%) of 22 eyes with outer nuclear layer cysts, whereas in only 1 of 14 eyes (7.1%) without outer nuclear layer cysts (p=0.009). Disorganization of retinal inner layers was present in 15 of 22 (68.1%) eyes with outer nuclear layer cysts, whereas only 2 of 14 (14.2%) without outer nuclear layer cysts had disorganization of retinal inner layers (p=0.013).

**Conclusion:**

Characteristics of intraretinal cystoid spaces may predict prognosis in patients with diabetic cystoid macular edema, and visual gain may be limited in the eyes with outer nuclear layer cysts.

## Abstract (pt)

**Objetivo:**

Avaliar se as características da tomografia de coerência óptica de domínio espectral dos espaços cistoides intraretinianos prevêem resultados visuais em pacientes que recebem terapia de injeção intravítrea com fator de crescimento endotelial antivascular (bevacizumab 1,25 mg/0,05 ml) para edema macular cistoide diabético.

**Métodos:**

A relação entre as propriedades dos espaços cistoides antes da injeção e os resultados anatômicos e funcionais após a injeção foi investigada em pacientes que receberam três injeções intravítreas para edema macular cistoide. A melhor acuidade visual corrigida para a melhora funcional e a espessura do subcampo central para a melhora anatômica foram avaliadas. Além disso, foi avaliada a relação da localização dos espaços cistoides com a integridade dos fotorreceptores e camadas internas da retina.

**Resultados:**

Em 36 olhos de 36 pacientes, a melhor acuidade visual corrigida foi significativamente aprimorada (p=0,002), e a espessura média do subcampo central foi diminuída após injeções (p=0,003). O aprimoramento da melhor acuidade visual corrigida foi limitado nos olhos com cistos na camada nuclear externa (p=0,045). A reflexividade intracística foi maior nos olhos que falharam na melhor acuidade visual corrigida do que nos olhos com resultados visuais bem-sucedidos (p=0,028). A zona elipsoide interrompida esteve presente em 13 (59,0%) de 22 olhos com cistos na camada nuclear externa, e em apenas 1 de 14 olhos (7,1%) sem cistos na camada nuclear externa (p=0,009). A desorganização das camadas internas da retina esteve presente em 15 dos 22 olhos (68,1%) com cistos na camada nuclear externa, enquanto apenas 2 em 14 olhos (14,2%) sem cistos na camada nuclear externa tiveram desorganização das camadas internas da retina (p=0,013).

**Conclusão:**

Características dos espaços cistoides intrarretinianos podem prever prognóstico em pacientes com edema macular cistoide diabético e ganho visual pode ser limitado nos olhos com cistos na camada nuclear externa.

## INTRODUCTION

Diabetic macular edema (DME) is the most important cause of visual loss in patients with diabetic retinopathy (DR)^(1,2)^. Laser photocoagulation had been the gold standard in the treatment of DME until the early 2000s^(3)^. Visual stabilization was aimed rather than an improved vision by administration of photocoagulation. After the intravitreal introduction of antivascular endothelial growth factor (anti-VEGF), it has been possible to achieve significant visual gains in patients with DME^(4-6)^. However, certain patients who received anti-VEGF treatment are resistant to treatment or have an inadequate response. In the Diabetic Retinopathy Clinical Research Network (DRCR.net) study, following ranibizumab treatment with either rapid or delayed laser, the central subfield thickness (CST) was ≥250 µm in 40% of the patients in 2 years^(7)^. In the light of these data, it may be argued that not all eyes respond to anti-VEGF therapy at the same level, and some predictive factors can guide the response to treatment in patients who will be treated with intravitreal injection of anti-VEGF.

Anatomical markers such as ellipsoid zone (EZ) damage and disorganization of retinal inner layers (DRIL) through spectral-domain optical coherence tomography (SD-OCT) are important in visual prognosis, while findings such as hyperreflective foci (HRF) and serous retinal detachment can guide the degree of inflammatory burden^(8)^. Morphological and topographic features of foveal cysts can help estimate treatment response^(9,10)^. The presence of foveal cysts in the outer layers of the retina indicates that the disease is more chronic, and the prognosis in these eyes is worse than in eyes with cysts concentrated in inner layers^(11,12)^. The density of the fluid within cystoid spaces can provide an indication of the fluid content and the severity of the blood-retina barrier disorder^(13)^. Taut posterior hyaloid membrane (TPHM) detected through SD-OCT is another reason for recalcitrant macular edema.

With the above background, this study aimed to evaluate the relationship between baseline SD-OCT characteristics of the foveal cystoid spaces and anatomical and visual outcomes after three consecutive bevacizumab (1.25mg) applications.

## METHODS

In the present study, 105 patients who have received only three consecutive bevacizumab treatments due to diabetes-related cystoid macular edema (CME) between April 2019 and September 2020 in Balıkesir University Faculty of Medicine were retrospectively evaluated. Patients with type II diabetes mellitus (DM) aged 40-60 years with CME in the foveal center were included in the study. The included patients were not naive, but we only included patients who have not received an intravitreal injection in the last 3 months and with laser photocoagulation in the last 6 months. Of 105 patients, 13 who did not comply with the final examination date, which was determined as 28-35 days after three injections, 10 who had accompanying dry-type age-related macular degeneration, 16 who had poor quality images, 6 who underwent cataract surgery within the last 6 months, 8 who had type I DM, and 16 who had edema type that was not CME were excluded. Consequently, 36 eyes of 36 patients were included in the study. When CME was present in both eyes, the eye with thicker CST was analyzed. Three consecutive monthly bevacizumab (1.25 mg/0.05 ml, Altuzan, Roche, Switzerland) injections were given in the 36 eyes of 36 patients.

All procedures and measurements adhered to the tenets of the Declaration of Helsinki. The study protocol was approved by Balikesir University Ethics Committee. All participants provided written informed consent before study enrollment.

Clinical and SD-OCT findings at 1-3 days before starting injections were accepted as the baseline examination, and examinations performed at 28-35 days after three injections were accepted as the final examination. After comprehensive ophthalmic examinations, retinal sectional images were obtained using SD-OCT (Cirrus HD-OCT; Carl Zeiss Meditec, Dublin, CA). Vertical and horizontal retinal sectional images dissecting the fovea were acquired using 30-degree cross-hair mode, and 20-100 images were averaged to create better images. The central subfield thickness of Early Treatment Diabetic Retinopathy Study grid (mean retinal thickness within a 1-mm circle centering on the fovea) was determined. Two ophthalmologists (GSV and CG) evaluated qualitative findings, and a third specialist (EK) settled any disagreements.

**Figure 1:**
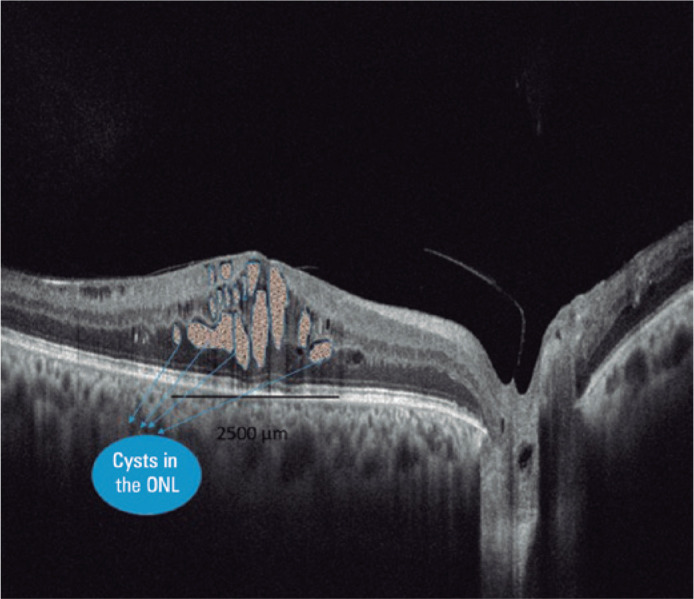
Cysts identified and marked in the area of 2500 µm; cysts in the outer retinal layer were also specified.

The best-corrected visual acuity (BCVA) was measured decimally at each visit and converted to the logarithm of minimum angle of resolution (logMAR) of the visual acuity. All SD-OCT images were imported into Image-J software (Fiji, NIH, Bethesda, MD, USA) for image processing^(14)^. All cysts were traced manually with the polygon tool option. The total area covered by the cystoid spaces in the area of 2500 micrometer diameter, including the fovea and parafovea, was measured (Figure 1). The baseline average reflectance values of the cystoid spaces were measured as described previously^(15)^. We further quantified the reflectivity levels of the vitreous cavity and nerve fiber layer as the internal standard in each image. The relative OCT reflectivity value of the cystoid spaces was calculated as an arbitrary unit according to the formula:

Relative reflectivity = Reflectivity (cystoid spaces) ­ reflectivity (vitreous) / Reflectivity (nerve fiber layer) ­ reflectivity(vitreous)

The mean reflectance value of all cysts was determined. The foveal photoreceptor status was manually evaluated according to a previous publication^(16)^. The status of the EZ line was classified into two categories, i.e., disrupted and intact. Eyes in which one or more boundaries between the layers of the ganglion cell-inner plexiform layer complex, inner nuclear layer, and outer plexiform layer were not separately identifiable were considered to have DRIL. Eyes with posterior hyaloid that form a sheet along the posterior pole, which resulted in tractional force and mechanical retinal distortion, were considered to have TPHM. Intraretinal dots that had a size of <30 µm, absence of back-shadowing, and reflectivity similar to the retinal nerve fiber layer was accepted as HRF.

Patients whose BCVA did not increase at least two lines after three injections were deemed to be unsuccessful in terms of vision, whereas those who had increased BCVA by more than two lines were considered successful regarding functionally. Patients whose central retinal thickness (CRT) value was ≤250 µm at the end of three injections were considered anatomically successful and >250 µm were considered unsuccessful.

Data were analyzed in IBM SPSS Statistics for Windows, version 22 (IBM Corp., Armonk, NY, USA). BCVA was transformed into logMAR for statistical analysis. Quantitative and qualitative data were presented by mean ± standard deviation (SD) and number or percentage, respectively. The relationship between pre-injection parameters and post-injection anatomical and visual outcomes was evaluated using binary and linear multiple regression analyses. Comparisons were conducted using the independent t-test for non-parametric data between anatomically and visually successful and unsuccessful eyes, and the chi-square test for parametric values. P<0.05 was considered significant.

## RESULTS

The baseline characteristics of the patients are shown in Table 1. The mean BCVA improved from 0.55 ± 0.29 to 0.40 ± 0.27 at the final visit (p=0.002). The mean CRT thickness decreased from 494.8 ± 152.1 µm to 370.8 ± 120.7 µm after three injections (p=0.003).

**Table 1:** Baseline characteristics, baseline and final BCVA, and CST

**Parameter**	
Eyes/patients	36/36
Age (years)	62.1 ± 9.3 (44-85 years)
Men/women	19/17
HbA1c (%)	7.3 ± 0.4 (6.6-8.0)
Systemic hypertension (no. of patients)	17
LogMAR VA	0.55 ± 0.28 (1.0-0.0)
Moderate nonproliferative DRP	12 eyes (33.3%)
Severe nonproliferative DRP	14 eyes (38.9%)
Proliferative DRP	10 eyes (27.8%)
Pseudophakia (%)	12 eyes (33.3%)
CSF thickness (µm)	494.7 ± 152.1
Hyperreflective foci in the inner retinal layers	24 eyes (66.6%)
Hperreflective foci in the outer retinal layers	17 eyes (47.2%)
Subretinal fluid	11 eyes (30.5%)
DRIL	18 eyes (50.0%)
Disrupted EZ line	16 eyes (44.4%)
Total cyst area	10946.5 ± 4014.6
Cystoid spaces in the outer retinal layers	21 eyes (58.3%)
Mean intracystic reflectance	43.3 ± 7.5

BCVA= best-corrected visual acuity; CST= central subfield thickness; DRP; DRIL= disorganization of retinal inner layers; EZ= ellipsoid zone; HbA1c= hemoglobin A1C.

At the final visit, 18 eyes (50.0%) had at least two lines of improvement in BCVA. There was no difference in the initial total cyst area between successful and unsuccessful eyes in terms of visual acuity (p=0.498). The improvement in visual acuity was more limited in eyes with a cystoid space in the outer nuclear layer (ONL) (p=0.015). Intracystic reflectivity was higher in eyes with poor BCVA than in eyes that had good vision after three injections (p=0.028). The rates of EZ defect and presence of DRIL were higher in the group with poor visual acuity (p=0.019, p=0.011, respectively). No difference was noted in the presence of TPHM, presence of subretinal fluid, and number of HRF between eyes with poor and better vision. Table 2 shows the comparison of the eyes with poor and better BCVA.

**Table 2:** Comparison of eyes according to the alterations in the final visual acuity.

	**Final BCVA improvement** **≥**2 logMAR lines (n=18)	**Final BCVA improvement** <2 logMAR lines (n=18)	**p-value**
Age	61.6 ± 9.9	62.5 ± 9.1	0.832
Gender			
Male (%)	58.3	50.0	0.682
Female (%)	41.7	50.0	
Time of DM (years)	10.6 ± 3.3	12.6 ± 1.7	0.063
HbA1c (%)	7.39 ± 0.38	7.32 ± 0.42	0.690
Baseline BCVA (logMAR)	0.60 ± 0.31	0.49 ± 0.25	0.328
Baseline CRT (µm)	525.6 ± 167.9	463.8 ± 134.4	0.331
Baseline total cyst area (µm^2^)	10102.5 ± 3316.3	11178.5 ± 4271.8	0.498
Baseline mean intracystic reflectance	40.5 ± 6.2	49.1 ± 7.9	0.028^*^
Hyperreflective dots (number)	13.6 ± 8.4	16.4 ± 9.3	0.443
Presence of DRIL			
Yes (%)	16.7	66.7	0.011^*^
No (%)	83.3	33.3	
Presence of subretinal fluid			
Yes (%)	44.4	38.8	0.346
No (%)	55.6	61.2	
Presence of cyst in outer retinal layer			
Yes (%)	83.3	41.7	0.015^*^
No (%)	16.7	58.3	
Presence of TPHM			
Yes (%)	27.8	50.0	0.145
No (%)	72.2	50.0	
Presence of disrupted EZ			
Yes (%)	38.8	77.7	0.019
No (%)	61.2	22.3	
Final CRT (µm)	222.5 ± 107.2	319.1 ± 117.8	0.047^*^
Final total cyst area (µm^2^)	910.2 ± 179.7	1890.8 ± 436.8	0.074

BCVA= best-corrected visual acuity; CRT, central retinal thickness; DM= diabetes mellitus; DRIL= disorganization of retinal inner layers; HbA1c= hemoglobin A1C; TPHM= taut posterior hyaloid membrane.

In 14 eyes, the final CRT was ≤250 µm, whereas in 22 eyes, it was >250 µm. None of the baseline parameters were different between eyes with final CRT ≤250 µm and with >250 µm (Table 3).

**Table 3:** Comparison of eyes with a final CRT value below and above 250 µm

	**Final CRT** **≤**250 **µ**m (n=15)	**Final CRT >**250 **µ**m (n=21)	**p-value**
Age	57.9,9 ± 8.9	64.6 ± 8.8	0.087
Gender			
Male (%)	53.3	52.4	0.455
Female (%)	46.7	47.6	
Time of DM (years)	10.2 ± 3.6	12.5 ± 1.7	0.101
HbA1c (%)	7.23 ± 0.44	7.43 ± 0.36	0.240
Baseline BCVA (logMAR)	0,60 ± 0.28	0.52 ± 0.29	0.519
Baseline CST (µm)	504.2 ± 190.1	489.1 ± 131.3	0.820
Baseline total cyst area (µm^2^)	10036.0 ± 3998.9	11003 ± 3736	0.556
Baseline mean intracystic reflectance	41.2 ± 6.3	44.5 ± 8.1	0.308
Hyperreflective dots	15.9 ± 9.2	14.5 ± 8.8	0.711
Presence of DRIL			
Yes (%)	40.0	42.8	0.322
No (%)	60.0	57.2	
Presence of subretinal fluid			
Yes (%)	33.3	47.6	0.386
No (%)	66.7	52.4	
Presence of cysts in outer retinal layer			
Yes (%)	77.8	53.3	0.225
No (%)	22.2	46.7	
Presence of disrupted EZ			
Yes (%)	33.3	42.8	0.208
No (%)	66.7	57.2	
Presence of TPHM			
Yes (%)	33.3	42.8	0.424
No (%)	66.7	57.2	
Final CRT (µm)	219.3 ± 24.2	339.4 ± 67.8	0.021^*^
Final total cyst area (µm^2^)	511.2 ± 134.3	1672.5 ± 239.5	0.004^*^

BCVA= best-corrected visual acuity; CST= central subfield thickness; CRT= central retinal thickness; DM= diabetes mellitus; DRIL= disorganization of retinal inner layers; HbA1c= hemoglobin A1C; TPHM= taut posterior hyaloid membrane.

Multiple linear regression analysis revealed that visual outcome was related to the baseline BCVA, presence of DRIL, presence of EZ defect, and presence of ONL cyst (Table 4).

**Table 4:** Correlation of the final BCVA with the baseline parameters

	**Final BCVA**	
**Parameters**	**R** ^2^	**p-value**
Age	0.107	0.118
Gender	0.357	0.819
Time of DM	0.012	0.940
HbA1c	0.138	0.074
Baseline BCVA (logMAR)	0.270	0.009^*^
Baseline CST (µm)	0.002	0.824
Baseline total cyst area (µm^2^)	0.084	0.466
Baseline mean intracystic reflectance	0.024	0.833
Hyperreflective dots	0.034	0.876
Presence of DRIL	0.292	0.014^*^
Presence of SRF	0.013	0.814
Presence of ONL cysts	0.321	0.004^*^
Presence of disrupted EZ	0.289	0.08^*^
Presence of TPHM	0.021	0.922

BCVA= best-corrected visual acuity; CRT= central retinal thickness; DM= diabetes mellitus; DRIL= disorganization of retinal inner layers; HbA1c= hemoglobin A1C; ONL= outer nuclear layer; SRF= subretinal fluid; TPHM= taut posterior hyaloid membrane.

Of the 36 eyes, 22 had cysts in the ONL. While 13 (59.0%) of 22 eyes with a cystoid space in the ONL had EZ disorder, only 1 (7.1%) of 14 eyes without cystoid space in the ONL had EZ disorder (p=0.009). In eyes with cysts in the ONL, the presence of DRIL was noted in 15 of 22 eyes (68.1%), 2 of 14 (14.2%) eyes without ONL cysts had DRIL (p=0.013).

## DISCUSSION

Imaging biomarkers have been a hot topic in recent years. Almost histological evaluation of changes in the retina is provided through these markers^(17)^. DRIL was found to be associated with the severity of maculopathy, especially the level of ischemic maculopathy^(18)^. DRIL is also more common in eyes with more severe signs of proliferative DR^(19)^. The robustness of EZ provides information about the health of the photoreceptor layer. As the degree and duration of DME increase, a more severe EZ impairment is encountered^(20)^. The response to anti-VEGF treatment is worse in eyes with TPHM^(21,22)^. The presence of hyperreflective foci and subretinal fluid is considered mostly associated with the level of inflammation. The present study revealed that visual outcomes were worse in eyes with DRIL, disrupted EZ, and eyes with cysts in the ONL treated with bevacizumab for CME.

The reflectivity of the cyst is believed to be determined by the level of plasma, blood, hyaline, fibrinous material, or macrophage inside the cysts^(13,16,23)^. Nevertheless, it is not known for certain what is responsible for the reflectance within the cysts. Fibrin or other inflammatory material might be responsible for intracystic reflectance^(23)^. In this study, the level of intra-cyst reflectance was higher in eyes with visual failure. It can be thought that DME has been present for a long time or inflammation is more severe in eyes with high intracystic material density. This may explain the relationship between visual failure and hyperreflective cysts after anti-VEGF injection.

Large ONL cysts are seen in the later stages of DME and negatively affect macular function^(24)^. In this study, a relationship was noted between the extension of cystoid spaces and photoreceptor damage. In addition, no relationship was found between the size of the cyst and visual results, but visual success was low in patients with ONL cysts. EZ damage was more extensive in eyes with ONL cysts. The rate of DRIL was also higher in eyes with ONL cysts. Is this effect of cysts caused by mechanical compression? Or are there other underlying pathophysiological mechanisms? The cystoid spaces primarily start in the inner layers, so the presence of an ONL cyst can give information about the onset of the pathology. Here, the negative effect of long-term edema on Muller cells, as a result of the disrupted glutamate uptake by Muller cells, and the neurotoxic effect of extracellular glutamate toxicity should be questioned. Accordingly, the rate of DRIL in those with ONL cysts was higher than in those without external cysts. This implies that the presence of ONL cysts indicates further damage in the inner layers. To understand this issue better, the presence of a relationship between the characteristics of cysts in eyes with cystoid formation and the health of intraretinal neurons should be examined.

This study has several limitations. First, the study has a small sample size and retrospective design. Second, due to the lack of information about anamnesis of the patients, no definite data could be obtained about the chronicity of CME. Third, since several SD-OCT findings were subjectively assessed in this study, future studies should develop procedures to evaluate them objectively. Fourth, examining the results of only three injections is a debilitating factor. More meaningful results can be obtained with a longer follow-up. Fifth, the fact that patients were not evaluated by OCT angiography and that those with severe ischemia were not excluded from the study reduces the power of the study. Sixth, the included patients were not naive, because most of the patients were referred to our hospital with a tertiary eye care center after initial treatment such as intravitreal injection or laser photocoagulation. Although the parameters would be affected by intraocular intervention, the inclusion of only näive patients may limit the number of cases for statistical analysis. We tried to overcome this by excluding patients who have received an intravitreal injection in the last 3 months, or with laser photocoagulation in the last 6 months. Despite all these shortcomings, we believe that our study is valuable in revealing the relationship between cysts in the ONL and the inner layers of the retina.

In conclusion, a very detailed examination of SD-OCT in patients who will be starting on intravitreal anti-VEGF therapy will provide very important data on the potential for response to treatment. Special attention should be paid to the localization and reflectance of intraretinal cysts, and visual success may be lower in patients with cysts with high reflectivity and cysts located on the outer retinal layers. More prospective studies with larger series are needed to determine customized therapies in patients with CME.
